# *Mycobacterium avium* complex peritonitis in a pediatric patient on peritoneal dialysis: A case report

**DOI:** 10.1097/MD.0000000000026321

**Published:** 2021-06-18

**Authors:** Shunsuke Yokota, Kentaro Nishi, Sho Ishiwa, Kazuhiro Uda, Kensuke Shoji, Koichi Kamei

**Affiliations:** a Division of Nephrology and Rheumatology; b Department of Pediatric Nephrology, Tokyo Women's Medical University; c Division of Infectious Diseases, National Center for Child Health and Development; d Division of Infectious Diseases, Tokyo Metropolitan Children's Medical Center, Tokyo, Japan.

**Keywords:** kidney transplantation, *Mycobacterium avium* complex, peritoneal dialysis, peritonitis

## Abstract

**Introduction:**

Peritonitis due to *Mycobacterium avium* complex (MAC) is a rare but life-threatening complication in patients on peritoneal dialysis (PD). However, optimal therapeutic regimen, treatment duration, and appropriate timing of kidney transplantation (KT) after treatment are unknown.

**Symptoms:**

We herein report a 4-year-old boy on PD due to end-stage kidney disease resulting from bilateral hypoplastic kidneys. He was admitted for peritonitis complaining fever, abdominal pain, and cloudy peritoneal effluent on PD after accidentally biting and opening the PD catheter while in the bath. Initial treatment with vancomycin and ceftazidime for 2 weeks was successful, although peritonitis recurred 37 days after discharge.

**Diagnosis:**

Mycobacterial culture was positive 9 days after readmission, and MAC was grown in the PD culture on day 30. We diagnosed him with MAC peritonitis that occurred on PD.

**Interventions:**

Clarithromycin, ethambutol, and rifampicin were initiated. The PD catheter was removed, and hemodialysis was initiated with a cuffed catheter inserted in the internal jugular vein. Follow-up observation for 8 months after the cessation of 1-year anti-mycobacterial therapy confirmed no recurrence of MAC infection, and the patient received living-donor KT from his father.

**Outcomes:**

His renal function was stable, with no recurrence of MAC peritonitis at 2 years after the KT.

**Conclusion:**

To the best of our knowledge, this is the first report of a patient who successfully underwent KT after receiving treatment for MAC peritonitis. One-year anti-mycobacterial therapy, PD catheter removal, 8-month observation after the cessation of therapy led the successful KT, although further investigation is warranted to confirm the efficacy of this approach.

## 1 Introduction

*Mycobacterium avium* complex (MAC) is a group of slowly growing mycobacteria and comprises >10 species including *Mycobacterium avium*, *Mycobacterium intracellulare*, *Mycobacterium indicus pranii*, *Mycobacterium chimaera*, *Mycobacterium arosiense*, *Mycobacterium vulneris*, *Mycobacterium boucheduhonense*, and *Mycobacterium colombiense*.^[1]^ Peritonitis due to MAC, albeit rare, is a severe complication that can become life-threatening for patients on peritoneal dialysis (PD).^[2,3]^ Recurrence of MAC infection can also be fatal after kidney transplantation (KT) in patients on immunosuppressive therapy. However, optimal therapeutic regimen, treatment duration, and appropriate timing of KT after anti-mycobacterial treatment for MAC infection are unknown, with no reports of patients who successfully received KT after treatment for PD-related peritonitis due to MAC infection.

Here, we present our experience with a pediatric patient who developed PD-related MAC peritonitis and received anti-mycobacterial treatment for 1 year while on hemodialysis; the patient subsequently underwent ABO-compatible living-donor KT without recurrence of MAC peritonitis.

## 2 Case report

A 4-year-old boy on PD due to end-stage kidney disease resulting from bilateral hypoplastic kidneys, was admitted to our hospital with the complaints of fever, abdominal pain, and cloudy peritoneal effluent on PD, 1 day after biting and accidentally opening the PD catheter while in the bath. He was planned to receive living related KT from his father.

His vital sign was the following: temperature of 37.0 °C, blood pressure of 96/48 mmHg, pulse of 144 beats per minute. On physical examination, he appeared fine and his extremities were not cold. There was no abdominal tenderness or other signs of peritoneal irritation. The catheter exit site was clear. Laboratory examination at admission showed the following: white blood cell count, 8180/μL; C-reactive protein, 3.17 mg/dL; and dialysate cell count, 775 /μL with 74% polymorphonuclear leukocytes. Although the peritoneal fluid culture was negative, he was clinically diagnosed with bacterial peritonitis and started on intraperitoneal vancomycin and ceftazidime. His condition improved promptly after admission, and his white blood cell count in the PD fluid returned to normal on day 7 after hospitalization. He was discharged after completing the 2-week antibiotic therapy.

He was readmitted due to fever 37 days after the discharge. His vital signs were as follows: temperature, 38.3 °C; blood pressure, 102/56 mmHg; and pulse, 112 beats/min. He had no abdominal pain, and the catheter exit site was clear. Abdominal computed tomography showed no evidence of encapsulating peritoneal sclerosis or abscess. However, due to a dialysate cell count of 1160/μL accompanied with persistent fever, the patient was started on intraperitoneal vancomycin and ceftazidime on day 5 after readmission, with consideration of relapse of bacterial peritonitis.

**Figure 1:**
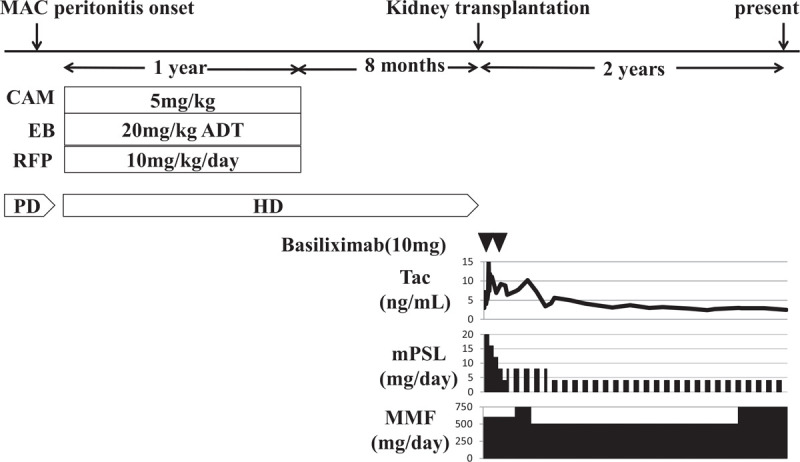
Clinical course of the patient. ADT = alternate-day treatment, CAM = clarithromycin, CCL = cefaclor, EB = ethambutol, HD = hemodialysis, MMF = mycophenolate mofetil, mPSL = methylprednisolone, PD = peritoneal dialysis, RFP = rifampicin, ST = trimethoprim-sulfamethoxazole, Tac = tacrolimus.

Although the bacterial and viral cultures of peritoneal fluid and blood culture were negative, the mycobacterial culture of peritoneal fluid became positive on day 9 after readmission. Interferon gamma release assay for tuberculosis and tuberculin test were negative, and atypical mycobacteria were suspected; therefore, the antibiotics were changed to clarithromycin, ciprofloxacin, and meropenem, considering rapid growing mycobacteria. The PD catheter was removed on day 19 after readmission, and hemodialysis was initiated using a cuffed 18-gauge catheter inserted in the internal jugular vein. Finally, *M avium* was identified on day 30, and we diagnosed him with MAC peritonitis that occurred on PD. The antibiotic therapy was changed to clarithromycin (5 mg/kg/d), ethambutol (20 mg/kg alternate-day treatment), and rifampicin (10 mg/kg/d) (Fig. 1). The patient's fever gradually improved, finally resolving in 1 month.

The 1-year anti-mycobacterial therapy was completed without any adverse events. We decided the plan of >6-month observation period after the treatment completion to confirm no recurrence of MAC peritonitis before proceeding to KT. He eventually received ABO-compatible living-donor KT from his father 8 months after the completion of anti-mycobacterial therapy. His post-transplant immunosuppressive regimen comprised methylprednisolone (mPSL), tacrolimus (Tac), and mycophenolate mofetil (MMF) (Fig. 1). His renal function was stable with a creatinine-estimated glomerular rate of 56.0 mL/min/m^2^ and no recurrence of MAC peritonitis at last follow-up 2 years after the KT.

## 3 Discussion

In this report, we presented the case of a 4-year-old boy on PD who developed MAC peritonitis successfully treated with anti-mycobacterial therapy and following received KT. We conducted anti-mycobacterial therapy for 1 year and observed the patient for 8 months after the cessation of anti-mycobacterial therapy before initiating KT. The patient did not experience recurrent MAC peritonitis during the 2-year follow-up period after KT while receiving the standard immunosuppressive regimen including mPSL, TAC, and MMF.

MAC peritonitis is a rare but highly lethal complication in patients with end-stage kidney disease on PD. Peritonitis due to nontuberculous mycobacteria is caused by MAC in around 10% of patients, with high mortality rates of up to 50%.^[2,4]^ Some patients with MAC peritonitis experience recurrence after treatment.^[2]^ To our knowledge, only 7 cases of MAC peritonitis were reported in patients on PD,^[3,5–11]^ with 4 mortalities, whereas no study to date has reported a patient who received KT after treatment for MAC peritonitis.

MAC organisms are ubiquitously found in various environmental conditions, including water and soil, and in animals. In the present case, the biting led to the opening of the PD catheter while the patient was in the bath. We predict that the patient likely had chemical peritonitis at first admission immediately after the incident due to the several reasons, including the very fast clinical onset and negative aerobic and anaerobic cultures of the peritoneal fluid. In contrast, the second peritonitis was caused by MAC, which was possibly present in the bath water.

Nontuberculous mycobacteria are classified into rapid and slow growers. On culture, the rapid growers form colonies within 7 days whereas the slow growers take more time. MAC are slow growing nontuberculous mycobacteria, and the colony formation took 8 days in the current case. In their review, Falcon et al^[3]^ reported that all 7 patients with MAC peritonitis presented with vague abdominal pain and that culture-negative peritonitis delayed diagnosis and treatment. Based on the current case, mycobacterial cultures should be performed for possible mycobacterial infection in patients whose clinical condition do not improve with empirical antibiotic therapy in the setting of negative standard bacterial culture of the peritoneal fluid.

In adult patients with extrapulmonary, localized MAC infections involving skin, soft tissue, tendons, joints, and bones, a combination of excisional surgery and chemotherapy is usually performed. Combination treatment with macrolides, ethambutol, and rifamycin is recommended by the American Thoracic Society and the Infectious Disease Society of America; however, optimal therapeutic regimens have not yet been established.^[2,12]^ In their systematic review, Xu et al^[13]^ reported that studies with treatment regimens containing macrolides had significantly higher success rates by pooled analysis (42%, 95% confidence interval 40–44%) compared with studies using other treatment regimens (28%, 95% confidence interval 24–32%), providing further evidence that macrolides are key therapeutic agents for MAC infections. On the other hand, the results of a randomized double-blind clinical trial found that resistance and treatment failure developed in almost half of the patients with MAC bacteremia who were treated with clarithromycin alone.^[14]^ Thus, multiple anti-mycobacterial therapy including macrolides is desirable. The optimal duration of treatment is unknown, but 6 to 12 months of therapy is usually recommended.^[11]^ Based on these facts, the current patient was treated with the combination of clarithromycin, ethambutol, and rifampicin for 1 year.

Immunosuppressive drugs after KT might be a risk factor for MAC infection recurrence, which can be life-threatening, or might otherwise cause rejection as the doses of immunosuppressive drugs are reduced during treatment for MAC infection.^[10,15]^ Although there was no available evidence on the appropriate duration between the cessation of anti-mycobacterial therapy and KT, we decided to observe our patient for a minimum of six months before KT to ensure the integrity of the treatment. After KT, the patient received the standard immunosuppressive protocol of mPSL, Tac, and MMF and did not experience recurrent MAC peritonitis for 2 years.

In conclusion, we herein presented the case of a 4-year-old boy on PD who developed MAC peritonitis and was successfully treated with 1-year anti-mycobacterial therapy; he successfully underwent KT after an 8-month interval after the cessation of treatment. Future studies of more cases are necessary to determined optimal therapeutic regimens, treatment duration, and appropriate time of KT after treatment for MAC peritonitis in patients on PD.

## Acknowledgments

The authors would like to thank Dr K. Ishikura and I. Miyairi for their clinical advice. They also like to thank all the staff of the Division of Nephrology and Rheumatology and the Division of Infectious Disease in the National Center for the Child Health and Development for their support of clinical practice. They also like to thank the patient and their guardians for providing clinical data for this study.

## Author contributions

**Conceptualization:** Sho Ishiwa.

**Methodology:** Kazuhiro Uda, Kensuke Shoji.

**Supervision:** Koichi Kamei.

**Writing – original graft:** Shunsuke Yokota.

**Writing – review & editing:** Kentaro Nishi, Kensuke Shoji, Koichi Kamei.
